# The high correlation between counts and area fractions of lipofuscin granules, a biomarker of oxidative stress in muscular dystrophies

**DOI:** 10.1007/s00418-016-1462-3

**Published:** 2016-07-09

**Authors:** Yoshiko Nakae, Peter J. Stoward

**Affiliations:** 1 Tokushima University Graduate School, Tokushima, Japan; 2 University of Dundee, Dundee, UK; 3 391 Kamigoryo Baba-cho, Kamikyo-ku, Kyoto, 602-0891 Japan

**Keywords:** Lipofuscin, Quantification, Image analysis, Skeletal muscle, *Mdx* mouse, Muscular dystrophy

## Abstract

Images of cryostat unstained sections of two skeletal muscles, diaphragm and extensor digitorum longus (EDL), from wild-type normal and dystrophic *mdx* mice were captured with a fluorescence microscope, binarised and analysed by an automated procedure using ImageJ free software. The numbers, Feret diameters and areas of autofluorescent lipofuscin (LF)-like granules in the sections were determined from the binary images. The mean numbers of counted LF granules per mm^3^ muscle tissue correlated highly (*r* ≥ 0.9) with the area fractions of the granules in sections of both normal and *mdx* muscles. The similar distribution patterns of granule sizes in sections of diaphragm and EDL muscles are consistent with the high correlations.

## Introduction

Previously, we and others have reported that lipofuscin (LF) granules are formed in large numbers in the dystrophin-deficient skeletal muscles of young *mdx* mice and of children with Duchenne muscular dystrophy, in contrast to their absence or sparseness in normal wild-type (WT) mice and healthy children (Nakae et al. 2001, 2004, 2008, 2012; Terrill et al. 2013; Tohma et al. 2011). The chemical composition of the LF-like granules in skeletal muscle and the details of the mechanism by which they are formed have not yet been unequivocally established. Nonetheless, we believe the granules are the products of oxidative stress (Nakae et al. 2008, 2012).

LF also accumulates in human myopathies other than Duchenne muscular dystrophy (Feeney et al. 2014; Terrill et al. 2013; Turki et al. 2012). Subsequently, we found that administration of an antioxidant polyphenol in green tea, epigallocatechin-3-gallate, to young dystrophic *mdx* mice significantly improved muscle function and reduced the mean numbers of autofluorescent LF granules per unit area in sections of diaphragm muscle of known thickness (Nakae et al. 2008, 2012). The amount of LF formed in skeletal muscles thus seems to be a promising quantitative biomarker of oxidative stress in muscular dystrophies.

However, we assumed in our previous reports that a count of fluorescent LF granules per unit area in a tissue section was a valid proxy for the fractional area or volume occupied by the granules and hence their concentration or amount, unlike assays of organic solvent extracts of autofluorescent constituents from tissues which show no correlation with in situ observations of LF (for example, by Sheehy 1996). With one possible exception (Tohma et al. 2011), there are no previous reports confirming that our assumption is valid. To address this issue, we have investigated the relationship of the two parameters, number counts and fractional areas, in sections of diaphragm and extensor digitorum longus (EDL) muscles of normal and dystrophic mice. The data reported here are consistent with our preliminary results published previously (Nakae et al. 2012): they confirm that there is a highly significant correlation (*r* ≥ 0.9) between the area fractions occupied by autofluorescent granules and their counts per unit tissue area or volume (called granule densities by Vila et al. 2000) despite variations in granule sizes. The distribution patterns of granule areas and Feret diameters in sections of skeletal muscle have also been investigated.

For the purpose of this paper, the autofluorescent granules observed in both normal and dystrophic muscle are called LF although it is widely asserted in the literature that whereas the autofluorescent granules present in normal ageing tissues are LF, those formed in pathological conditions consist of ceroid, which has a different chemical composition and tissue distribution from LF (Porta 2002; Porta et al. 2002; Yin 1996). However, as their number increases with age as the result of oxidative stress in the same way as occurs in normal ageing muscle (Nakae et al. 2004; Terrill et al. 2013), the autofluorescent granules formed in dystrophic muscle appear to be more akin to LF than ceroid.

## Materials and methods

Sections of diaphragm and EDL muscles and their autofluorescent images prepared for our previous study (Nakae et al. 2012) were used. The left costal part of the diaphragm muscle and left EDL muscle were removed by blunt dissection from 8-week-old male dystrophic C57BL/10-*mdx* (*mdx*) mice and age-matched male wild-type (WT) C57BL/10 normal mice fed with standard laboratory rodent diet pellets. Eight *mdx* diaphragm samples, 10 *mdx* EDL samples and 11 samples of the corresponding muscles of WT normal mice were used.

Cryostat unfixed transverse sections, 7-μm-thick, of the muscles were prepared as described previously (Nakae et al. 2012). One section was chosen from approximately the same position of each muscle and used for measuring the number, areas and Feret diameters of autofluorescent granules. The section was mounted in a mixture of 13.3 % Mowiol 4–88 Reagent (CalBiochem, San Diego, CA, USA) and 33.3 % glycerol (Fluka, Buchs, Switzerland) in 0.133 M Tris-HCl buffer, pH 8.5, kept at 4 °C for 1 day in the dark and then stored at −80 °C until used within 3 days after mounting for the fluorescence measurements.

### Image capture

Emission signals at 515 nm from the section excited at 450–490 nm were captured as 30–70 images for each section of diaphragm and 5–16 images for each EDL muscle section using a Spot Insight B/W camera (model 3.1, Visitron Systems, Puchheim, Germany) fitted to a fluorescence microscope with a ×20 objective (Zeiss Axiovert 200 M; Carl Zeiss MicroImaging, Jena, Germany). MetaView software (Visitron Systems) was used for the image capture.

### Image analysis

**Fig. 1:**
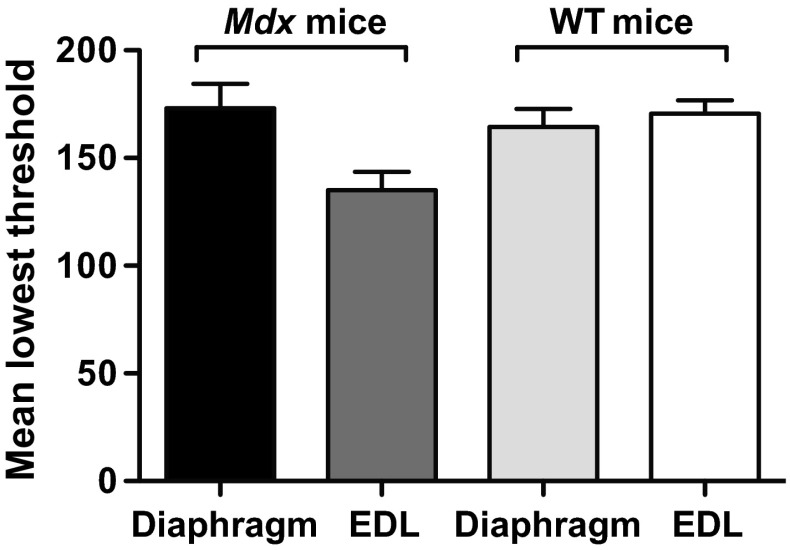
Means ± SEM of the lowest threshold levels used for the binarisation of autofluorescent images of two muscles in *mdx* and WT normal mice. 8 and 10 samples of *mdx* diaphragm and EDL muscles, respectively, and 11 and 10 samples of the corresponding muscles in WT normal mice were used

The total number of autofluorescent granules per image, the area and Feret diameter of each granule, and the whole section surface area analysed were determined by an automated procedure on every captured image of all the muscle sections using ImageJ free software version 1.41o (NIH, Maryland, USA). Each autofluorescence image was first converted to an 8- or 16-bit image, then binarised and the threshold pixel intensity range adjusted to capture the fluorescent granules. The maximum threshold level was set at 255 (2^8^–1), which corresponds to a completely white pixel. The lowest threshold level corresponded to the point where the granules were just discriminated from the (black) background and was adjusted to extract similar numbers of granules to those obtained manually from the raw image. The mean lowest threshold levels required for the four muscles studied are summarised in Fig. 1. The same results (lowest threshold, granule counts, areas and Feret diameters) were obtained with 8- and 16-bit images. The edges of the section and artefacts were excluded from the analysis. After setting the range of particle size to 1.50 µm^2^ to infinity and the granule circularity to 0.00–1.00, the area and Feret diameter of each granule were measured in the binary image. The minimum area of the particle corresponds to an assumed circle with a diameter of 1.38 μm. This size was chosen as it was close to the minimum size (diameter about 1 μm) of the granules that can be counted easily in the image as seen by the naked eye. The total number of granules per mm^3^ of muscle (also called granule density) was calculated assuming that the muscle section was uniformly 7-μm-thick. The relative area occupied by the granules was expressed as an area fraction (%) of the whole surface area analysed. For more precise measurements of the number of granules and their sizes, a black line with 1-pixel width was drawn between two close abutting granules in the original autofluorescence image if they were recognised as separate granules with the naked eye.

### Data analyses

GraphPad Prism software version 5.0c (GraphPad Software, La Jolla, CA, USA) was used for all data analyses. The correlations of granule densities (number of granules/mm^3^) with area fractions (%) of autofluorescent deposits in WT normal and *mdx* muscle were estimated as Pearson product-moment correlation coefficients (*r*). If there was a high correlation between two variables, linear regression best-fit and 95 % confidence limits were obtained from *x*–*y* plots of the variables. Further, tests of equalities among the three slopes (regression coefficients) and three *y*-intercepts of the regression lines for the different muscles analysed were performed using a comparison function in the software.

The relative frequencies (%) of the cumulative distribution (Downey 2014) of the sizes, areas in μm^2^ and Feret diameters in μm, of autofluorescent granules were determined in normal and *mdx* muscles and plotted against, respectively, mean areas and mean Feret diameters of the granules on a logarithmic scale.

A two-tailed unpaired Student’s *t* test was used to assess statistical significances between two means.

## Results

### Images of autofluorescent LF granules

**Fig. 2:**
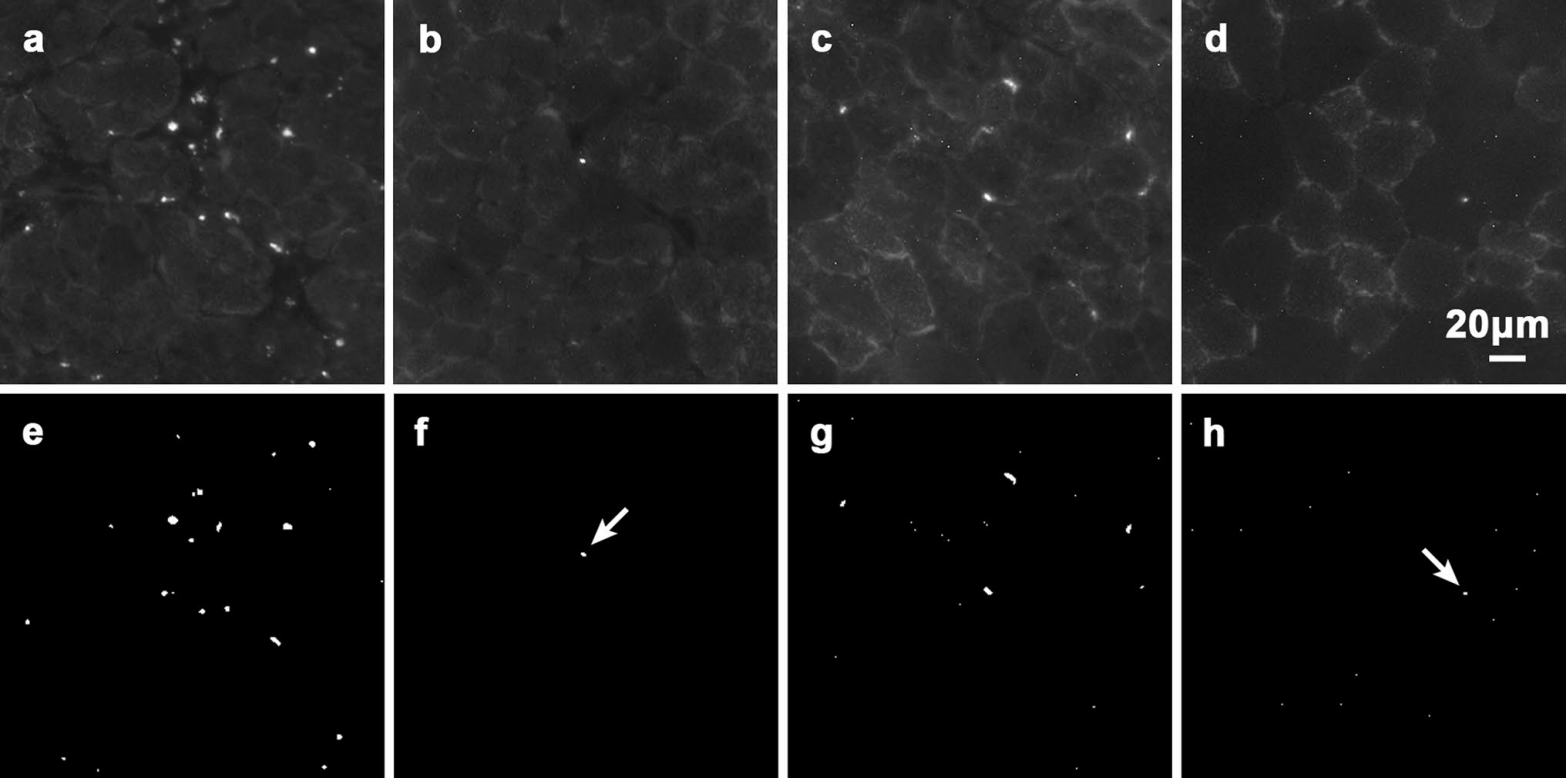
Typical raw images of autofluorecent LF granules in 7-μm-thick sections of diaphragm muscles of **a** 8-week-old *mdx* mice and **b** WT normal mice and EDL muscles of **c** *mdx* mice and **d** WT normal mice. Their corresponding binary images are shown in (**e**–**h**). As a measure of the granule sizes, two extracted LF granules (*arrows*) are shown in (**f**, **h**): the granule areas are 5.07 and 3.04 μm^2^, respectively, and their Feret diameters 3.56 and 2.57 μm, respectively

All raw images of autofluorescent granules in sections of WT normal and *mdx* muscles were obtained under the same condition as those reported previously (Nakae et al. 2012). Typical raw images and their binarised images are shown in Fig. 2. The LF granules in *mdx* muscles were mostly localised to the necrotic areas as reported previously (Nakae et al. 2004). They were more abundant in diaphragm muscles than EDL muscles (Fig. 2a, c; Nakae et al. 2012). In contrast, the granules were far fewer in WT normal muscles (Fig. 2b, d). As reported previously, LF granules were present in muscle fibres, myosatellite cells and interstitial cells (Nakae et al. 2001, 2004). Since the autofluorescence intensities of most LF granules were much higher than those of the structures surrounding them (such as myofibrils in the muscle fibres and ground substance in the connective tissue), they could be extracted by binarisation of the images (Fig. 2e–h). Only granules with areas ≥1.50 μm^2^ were analysed in the binary images.

### Parameters of LF granules

Table 1 records the mean ± SEM of the areas (μm^2^) and Feret diameters (μm) of LF granules in sections of 8-week-old normal and age-matched *mdx* muscles. This table also includes the mean ± SEM of the granule densities (numbers/mm^3^ tissue) and area fractions (%) of the granules, as measures of the relative amounts of the lipopigments granules present in the muscles. Student’s *t* test showed no significant differences in either the mean sizes or the amounts of the granules in normal diaphragm compared to EDL muscle (Table 1). The mean sizes (areas) of the granules in both *mdx* diaphragm and EDL muscles were nearly twice those (*P* < 0.0001) in the corresponding normal muscles (Table 2). The mean Feret diameters of the granules in *mdx* muscles were about 15–30 % higher (*P* < 0.05) than those in normal muscles. Although there were no significant differences in the sizes and amounts of LF granules in normal diaphragm and EDL muscles, the mean granule density and area fraction of the granules in *mdx* diaphragm muscle were, respectively, sevenfold (*P* < 0.0001) and ninefold (*P* < 0.0001) higher than those in *mdx* EDL muscle. However, the most notable difference between the *mdx* and normal muscles is the number density and area fraction of the granules, 50–100 times more in diaphragm muscle and 7 to about 14 times more in EDL muscle (Table 2).

**Table 1:** Means ± SEM of four parameters of autofluorescent LF granules in sections of two muscles of 8-week-old normal and *mdx* mice

Parameter	LF granules in normal		LF granules in *mdx*		Student’s *t* test			
	(a) Diaphragm (*n* = 11)	(b) EDL (*n* = 10)	(c) Diaphragm (*n* = 8)	(d) EDL (*n* = 10)	a versus b	c versus d	a versus c	b versus d
Granule size (µm^2^)	4.83 ± 0.33	4.31 ± 0.42	9.85 ± 0.55	7.72 ± 0.38	ns	**	****	****
Feret diameter (μm)	3.56 ± 0.13	3.57 ± 0.17	4.66 ± 0.10	4.10 ± 0.10	ns	**	****	*
Number of granules/mm^3^ tissue	1310 ± 270	1260 ± 270	64,900 ± 4600	9,320 ± 760	ns	****	****	****
Area fraction of granules (%)	0.00464 ± 0.00117	0.00406 ± 0.00098	0.456 ± 0.052	0.0508 ± 0.0051	ns	****	****	****

*n* = number of muscle samples

Significances of differences *P*: **** *P* < 0.0001; *** 0.0001 ≤ *P* < 0.001; ** 0.001 ≤ *P* < 0.01; * 0.01 ≤ *P* < 0.05; *ns* not significant

**Table 2:** Ratios of mean parameters of autofluorescent LF granules in two *mdx* and normal muscles in 8-week-old mice calculated from data in Table 1

Ratio of mean parameter for	Normal diaphragm/EDL	*Mdx* diaphragm/EDL	*Mdx*/normal diaphragm	*Mdx*/normal EDL
Granule size	1.12	1.28**	2.04****	1.79****
Feret diameter	1.00	1.14**	1.31****	1.15*
Granule count or density	1.04	6.96****	49.5****	7.40****
Granule area fraction	1.14	8.98****	98.3****	12.5****

Significances of differences *P*: **** *P* < 0.0001; *** 0.0001 ≤ *P* < 0.001; ** 0.001 ≤ *P* < 0.01; * 0.01 ≤ *P* < 0.05

### Relationship between granule density and area fraction of LF granules in normal muscles

**Fig. 3:**
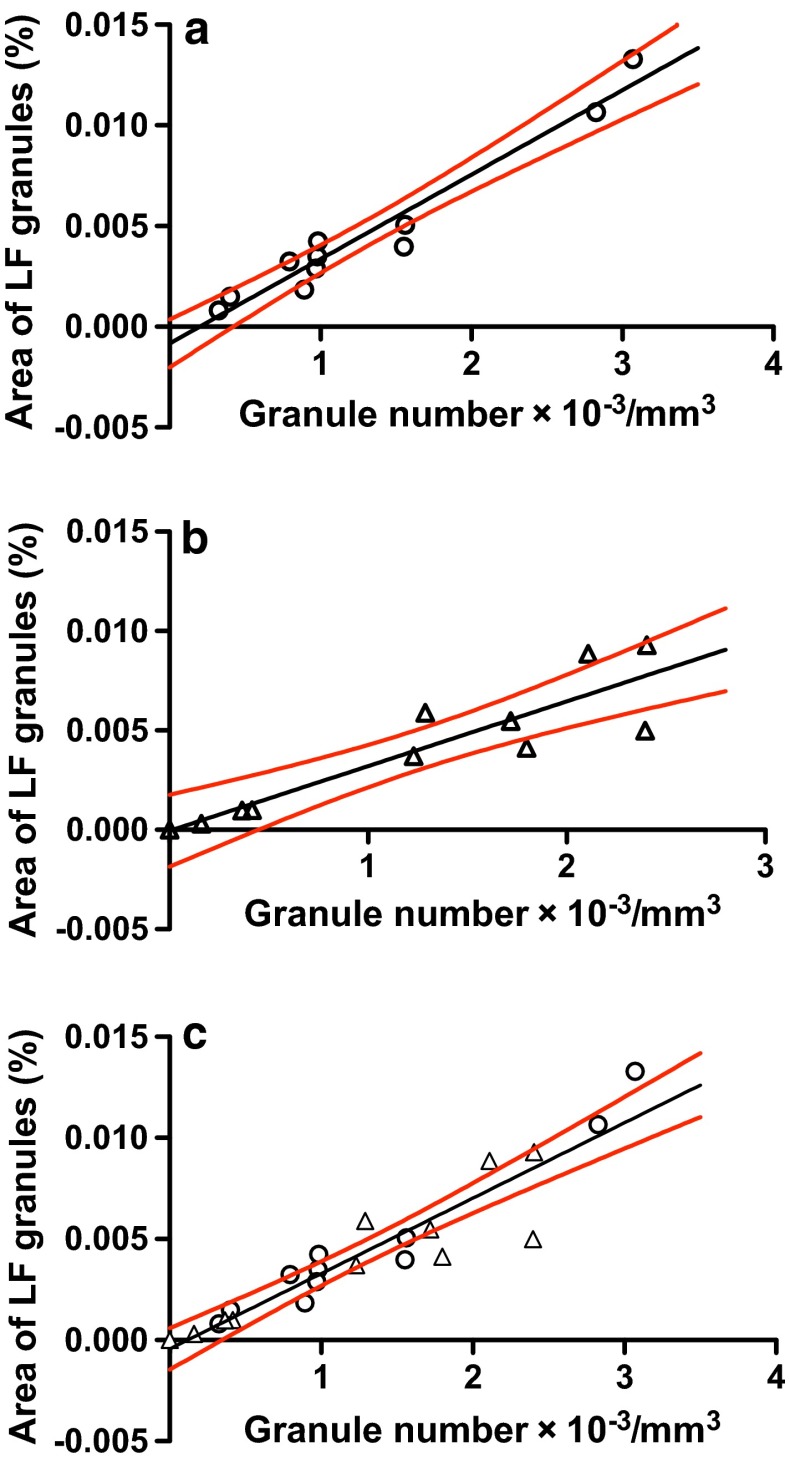
Correlations between area fraction (%) and number/mm^3^ tissue of LF granules in **a** diaphragm, **b** EDL and **c** diaphragm (*open circle*) plus EDL (*open triangle*) muscles of 8-week-old WT normal mice. Pearson correlation coefficient *r* = 0.972 (*P* < 0.0001, *n* = 11), 0.900 (*P* = 0.0002, *n* = 11) and 0.934 (*P* < 0.0001, *n* = 22) in (**a**–**c**), respectively. *n* = number of muscle samples. The *black* and *red lines* are, respectively, linear regression best-fits and 95 % confidence limits (see text for details)

Figure 3 shows plots of area fraction (%) on the *y* axis against granule density (number of granules/mm^3^) on the *x* axis for LF granules in (a) normal diaphragm (*n* = 11) and (b) normal EDL (*n* = 11) muscle and (c) the combined plots (*n* = 22) of (a) plus (b). The Pearson correlation coefficients (*r*) of the plots obtained for normal diaphragm, EDL, and diaphragm plus EDL muscles were high and significant, respectively, 0.972 (*P* < 0.0001), 0.900 (*P* = 0.0002) and 0.934 (*P* < 0.0001), indicating high correlations between the two parameters. The equations for the best-fit linear regression plots were *y* = (4.20 × 10^−6^ ± 3.38 × 10^−7^)*x* − (0.000835 ± 0.000527), *y* = (3.25 × 10^−6^ ± 5.24 × 10^−7^)*x* − (5.03 × 10^−5^ ± 0.000801) and *y* = (3.73 × 10^−6^ ± 3.19 × 10^−7^)*x* − (0.000439 ± 0.000492) for WT normal diaphragm, EDL and diaphragm plus EDL muscles, respectively. The three regression lines passed near through the origins. Comparisons of the three slopes and *y*-intercepts, using GraphPad Prism software, showed that the variance ratio (*F*) = 1.11, the degree of freedom numerator (*dfn*) = 2, degree of freedom denominator (*dfd*) = 38 and probability (*P*) = 0.340 for the slopes and *F* = 0.303, *dfn* = 2, *dfd* = 40 and *P* = 0.740 for the *y*-intercepts. This indicates that there are no significant differences among three slopes and among three intercepts. Pooled or common slope and *y*-intercept were calculated to be 3.73 × 10^−6^ and −0.000435, respectively, for three sets of data. These values almost coincide with those of the regression best-fit calculated for the combined data of WT normal diaphragm and EDL muscles (Fig. 3c).

### Relationship between granule density and area fraction of LF granules in *mdx* muscles

**Fig. 4:**
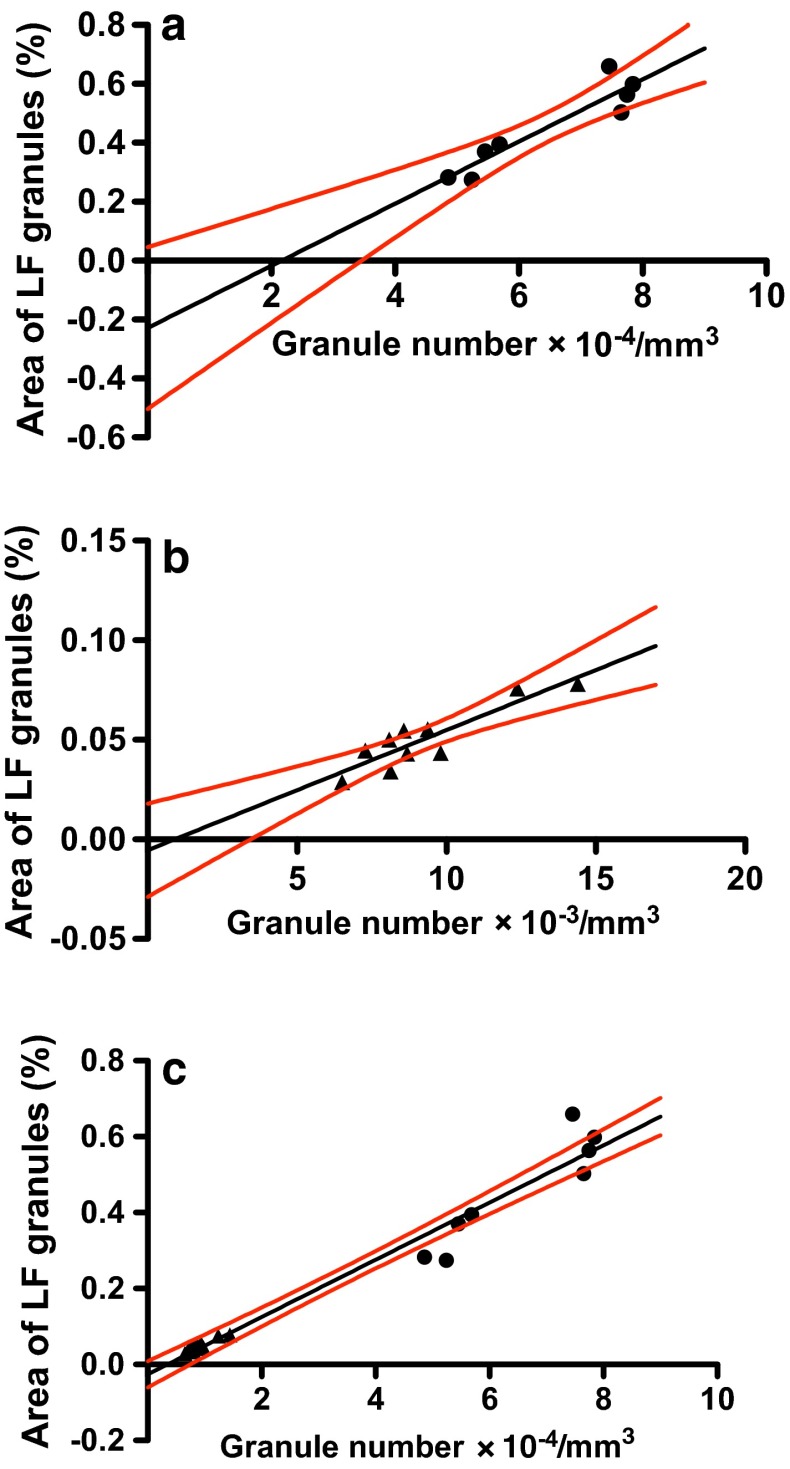
Correlations between area fraction (%) and number/mm^3^ tissue of LF granules in **a** diaphragm, **b** EDL and **c** diaphragm (*filled circle*) plus EDL (*filled triangle*) muscles of 8-week-old dystrophic *mdx* mice. Pearson correlation coefficient *r* = 0.930 (*P* = 0.0008, *n* = 8), 0.896 (*P* = 0.0005, *n* = 10) and 0.982 (*P* < 0.0001, *n* = 18) in (**a**–**c**), respectively. *n* = number of muscle samples. The *black* and *red lines* are, respectively, linear regression best-fits and 95 % confidence limits (see text for details)

Figure 4 shows plots of area fraction (%) on density (number of granules/mm^3^) for LF granules in (a) *mdx* diaphragm muscle (*n* = 8), (b) *mdx* EDL muscle (*n* = 10) and (c) the combined plots (*n* = 18) of (a) plus (b). The high *r* values, 0.930 (*P* = 0.0008), 0.896 (*P* = 0.0005) and 0.982 (*P* < 0.0001) found, respectively, for *mdx* diaphragm, EDL and diaphragm plus EDL muscles, indicated strong correlations between the two parameters. The linear regression best-fits were *y* = (1.05 × 10^−5^ ± 1.70 × 10^−6^)*x* − (0.229 ± 0.112), *y* = (6.03 × 10^−6^ ± 1.06 × 10^−6^)*x* − (0.00543 ± 0.0102) and *y* = (7.54 × 10^−6^ ± 3.66 × 10^−7^)*x* − (0.0256 ± 0.0163) for *mdx* diaphragm, EDL and diaphragm plus EDL muscles, respectively. The slopes and *y*-intercepts were not significantly different among the three regression lines (*F* = 2.82, *dfn* = 2, *dfd* = 30 and *P* = 0.0758 for the slopes; *F* = 0.402, *dfn* = 2, *dfd* = 32 and *P* = 0.672 for the *y*-intercepts). Pooled slope and *y*-intercept calculated for three sets of data were found to be 7.75 × 10^−6^ and −0.0328, respectively. These values were very similar to those of the regression best-fit for the combined data of *mdx* diaphragm plus EDL muscles (Fig. 4c).

### Distribution of sizes of LF granules in muscles

**Fig. 5:**
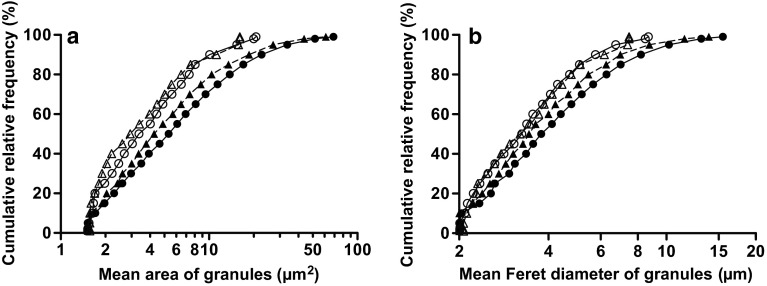
Cumulative distribution functions of the mean **a** areas and **b** Feret diameters of single LF granules in 8-week-old normal (WT) and *mdx* muscles. Each point is the mean for the following numbers of samples: *n* = 11 and 10, respectively, for normal diaphragm (*open circle*) and EDL (*open triangle*) muscles and 8 and 10 for *mdx* diaphragm (*filled circle*) and EDL (*filled triangle*) muscles, respectively

The cumulative frequency function estimates the probability that observed values are less than or equal to a specified value (Downey 2014). It is useful for examining whether the distribution of granule sizes is equal or not in the same or different types of muscles. Figure 5 shows the cumulative relative frequency (%) plotted on (a) mean area (µm^2^) and (b) mean Feret diameter (µm) of single LF granules in WT normal and *mdx* muscles. The mean ± SEM of the areas (µm^2^) of single granules at several representative relative frequencies in Fig. 5a and Student’s *t* test between two means are shown in Table 3. In Fig. 5, the curves connecting experimental points for LF granules in WT diaphragm and EDL muscles nearly overlapped. However, the corresponding curves for the mean area of LF granules in *mdx* diaphragm and EDL muscles shifted to larger area values by about 2.0 and 1.3 µm^2^, respectively, at the cumulative relative frequency 50 % (Fig. 5a; Table 3). These results were corroborated by the observed statistical distribution of the Feret diameters of the granules: the corresponding curves for the mean Feret diameter of the granules in *mdx* diaphragm and EDL muscles shifted similarly to longer diameter values by about 0.5 and 0.2 µm, respectively, at the 50 % cumulative relative frequency level (Fig. 5b). The mean area of the granules in *mdx* diaphragm muscles was similar to that in *mdx* EDL muscles at cumulative relative frequencies lower than 25 %, but at relative frequencies of 50–90 % the mean area was significantly different from that in *mdx* EDL muscles and their ratios (diaphragm/EDL) were in the range of 1.22–1.30 (Table 3).

**Table 3:** Cumulative distribution of mean sizes (areas) ± SEM (μm^2^) of single autofluorescent LF granules in sections of two muscles of 8-week-old mice

Cumulative relative frequency (%)	LF granules in normal		LF granules in *mdx*		Student’s *t* test			
	(a) Diaphragm (*n* = 11)	(b) EDL (*n* = 10)	(c) Diaphragm (*n* = 8)	(d) EDL (*n* = 10)	a versus b	c versus d	a versus c	b versus d
1	1.52 ± 0.00	1.57 ± 0.05	1.52 ± 0.00 (1.00)^§^	1.52 ± 0.00	ns	ns	ns	ns
10	1.64 ± 0.09	1.57 ± 0.05	1.71 ± 0.09 (1.08)	1.59 ± 0.05	ns	ns	ns	ns
25	1.98 ± 0.12	1.81 ± 0.08	2.60 ± 0.11 (1.08)	2.41 ± 0.07	ns	ns	**	****
50	3.37 ± 0.22	2.94 ± 0.23	5.33 ± 0.32 (1.25)	4.26 ± 0.20	ns	**	****	***
75	6.49 ± 0.57	5.57 ± 0.42	11.4 ± 0.8 (1.30)	8.78 ± 0.50	ns	**	****	***
90	10.1 ± 1.1	11.2 ± 2.6	22.6 ± 1.5 (1.22)	18.6 ± 1.1	ns	*	****	*

*n* number of muscle samples

Significances of differences *P*: **** *P* < 0.0001; *** 0.0001 ≤ *P* < 0.001; ** 0.001 ≤ *P* < 0.01; * 0.01 ≤ *P* < 0.05; *ns* not significant

^§^Figure in parentheses is the ratio of the mean for *mdx* diaphragm to the corresponding mean for *mdx* EDL

## Discussion

Shortly before our last report (Nakae et al. 2012) of LF in normal and dystrophic muscle was published, Tohma et al. (2011) analysed the experimental factors that might influence the precision and sensitivity of automated counting methods for quantifying LF and ceroid granules in situ in skeletal muscle. In particular, they examined the effects of section thickness, photo-bleaching and the pixel size of images. They used a different statistical approach from the one we have used, and their results do not affect the data and conclusions we report here.

The ratios (auto/manual) of the mean counts of LF granules per unit area or volume that we obtained automatically from binary images (Table 1) to those of our previous measurements carried out manually on the same raw images (Nakae et al. 2012) are 0.819 and 0.768, respectively, for WT normal diaphragm and EDL muscles and 1.01 and 1.06, respectively, for the corresponding *mdx* muscles. The approximately 20 % lower automated counts of granules in WT normal muscles are within 1 SEM. This suggests that about 80–100 % of the granules were extracted from the binary images in the present study and allowed the correlation between the granule counts and area fraction of the granules to be determined in such binary images.

We report for the first time a high correlation between LF granule counts (number/mm^3^ muscle tissue) and the percentage area fractions occupied by the granules in sections of skeletal muscle. The relationship is approximated by straight lines, *y* = 3.73 × 10^−6^ *x* − 0.000435 and *y* = 7.75 × 10^−6^ *x* − 0.0328 for LF granules in normal and *mdx* muscles, respectively (Figs. 3, 4). The two valuables are proportional since the straight lines pass through near the origin.

Why are counts of LF granules per unit area of tissue proportional to their area fraction? If the area of each granule is the same in the sections, these variables should be theoretically completely correlated (*r* = 1) and proportional. We hypothesise that a similar distribution pattern of granule sizes in skeletal muscle is responsible for the proportional relationship. The sizes of LF granules as expressed by their mean area (Fig. 5a; Table 3) or their Feret diameters (Fig. 5b) are distributed in the almost same patterns in both normal and dystrophic diaphragm and EDL muscle. Therefore, our hypothesis seems to hold for LF granules in these muscles. However, the areas and Feret diameters of LF granules at cumulative relative frequencies between 50–90 % are significantly 22–30 % (Table 3) and 10–17 % larger, respectively, in dystrophic diaphragm muscle than those in EDL muscle. Such differences in granule sizes do not seem to have significant effects on the proportional relationship between counts per unit area and the area fraction of LF granules in these muscles (Fig. 4c).

In this study, we have demonstrated that granule counts of LF granules per unit area as well as the fractional area occupied by autofluorescent granules are valid measures of the relative amounts of LF-like substances in situ, at least in the skeletal muscles of mice. We see no reason why the valid relationship should not also hold for other tissues, whether healthy or diseased. Our study also suggests that cumulative distribution functions of the granule sizes are a sensitive measure of chronic oxidative stress and can be applied for the quantitative evaluation of the effects of drugs on such stress.
